# Biomechanics principle of elbow joint for transhumeral prostheses: comparison of normal hand, body-powered, myoelectric & air splint prostheses

**DOI:** 10.1186/1475-925X-13-134

**Published:** 2014-09-10

**Authors:** Nasrul Anuar Abd Razak, Noor Azuan Abu Osman, Hossein Gholizadeh, Sadeeq Ali

**Affiliations:** Department of Biomedical Engineering, Faculty of Engineering, University of Malaya, Kuala Lumpur, 50603 Malaysia

## Abstract

**Background:**

Understanding of kinematics force applied at the elbow is important in many fields, including biomechanics, biomedical engineering and rehabilitation. This paper provides a comparison of a mathematical model of elbow joint using three different types of prosthetics for transhumeral user, and characterizes the forces required to overcome the passive mechanical of the prosthetics at the residual limb.

**Methods:**

The study modeled the elbow as a universal joint with intersecting axes of x-axis and y-axis in a plain of upper arm and lower arm. The equations of force applied, torque, weight and length of different type of prosthetics and the anthropometry of prosthetics hand are discussed in this study. The study also compares the force, torque and pressure while using all three types of prosthetics with the normal hand.

**Results:**

The result was measured from the elbow kinematics of seven amputees, using three different types of prosthetics. The F-Scan sensor used in the study is to determine the pressure applied at the residual limb while wearing different type of prostheses.

**Conclusion:**

These technological advances in assessment the biomechanics of an elbow joint for three different type of prosthetics with the normal hand bring the new information for the amputees and prosthetist to choose the most suitable device to be worn daily.

## Background

The biomechanics of an elbow joint is an essential parameter in a kinematics model for estimating force and length from the movement and rotation of a joint which the muscle crosses [1, 2]. The mechanical attributes of the elbow complex are mirrored by complementary clinical problems: the large ranges of motion are subject to significant losses following trauma or arthritic degeneration; the stability of the joint, which depends on both osseous and soft tissue structures, may be compromised by trauma or sporting activities, and the strength of the patient in activities of daily life are all mechanical factors that affect the performance of the joint [1–4].

Some studies were focused on the bone structure [5], tissue and muscle [6–11], and kinematics joint of elbow [12–14] for upper limb part. There are also a lot of studies focusing on the flexion and extension of elbow from normal human hand biomechanics principle [15]. The force applied by three main muscles along the elbow should be greater than the force applied to the lower part of elbow [1–4, 6–12]. The tendency of elbow muscle holding humerus with ulna and radius bone will be different if compared to the elbow joint when using the prostheses [16–19].

Different transhumeral prostheses provide different kinematics of motion [2–4, 6, 9–11, 16]. Most of the upper elbow prostheses come with the elbow joint which is the origin of axis in this study. The biomechanics principle of three different types of prosthetics which are body-powered, myoelectric and air splint prostheses is the interest of this study.

**Figure 1:**
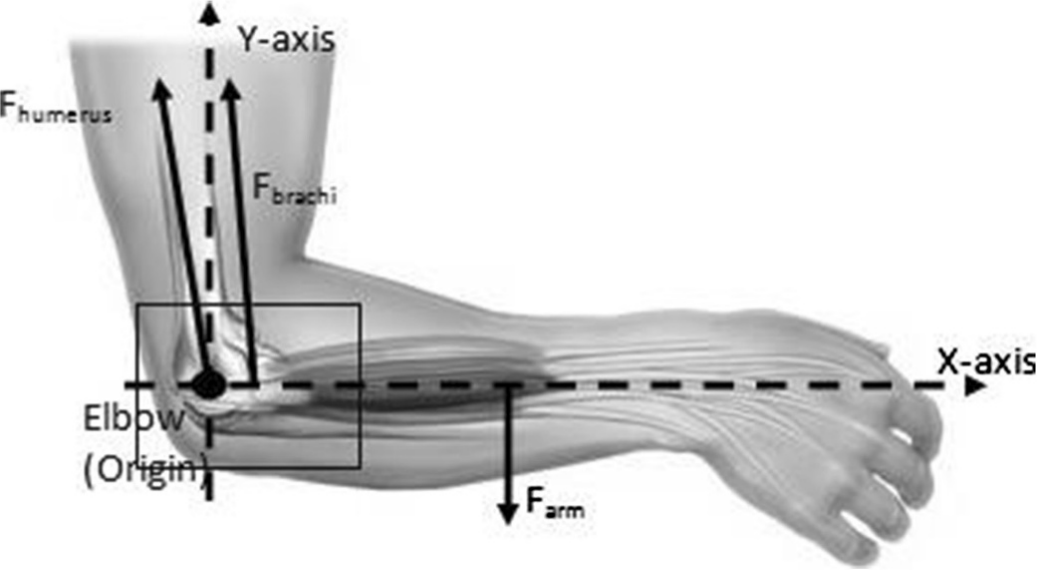
**Force exist as elbow become the origin.**

The muscle action is usually shown diagrammatically as only supplied by the biceps, and its tension (N) times the distance (m) of its line of action from the elbow axis gives the flexing moment (Nm), which opposes the extending moment of the weight in the hand [20–25]. Thus, taking typical approximate values, with the weight 350 mm from the elbow axis and a biceps tendon of 50 mm, rotational equilibrium of the moments acting about the flexion axis demands that the biceps tension must be equal seven times the external load (Figure 1). Further, with the load acting downwards and the biceps pulling upwards, parallel to the humerus, there is a net resultant force of six times the external load acting upwards onto the distal end of the humerus [1, 2, 4, 5, 21, 25, 26]. While this is a gross simplification, it does, nevertheless, show how the lever arm effect causes the internal forces to be multiples of the external loads.

It is widely known and accepted between amputees and prosthetists with a poor socket fit will entail the stump loses volume daily [2]. The amputee’s socket interface plays a major role in defining the comfort level of the user. Using the method which the socket is attached to the residual limb is extremely important [27–29]. Upper-extremity prostheses should be suspended throughout to the entire range of motion as well as being able to tolerate loading during normal use [28]. Furthermore, the amputees may need to change the socket in response to changes in body weight or alterations to the structure of the residual limb [16, 19, 30].

The body-powered prosthetic usually consists of a tension bowden cable, screw and joint, and socket [16]. The material used for the socket is the similar material that used to design the prototype of each individual case. These types of prosthetics usually follow the desired length of needed by referring to the other side of the hand that is not amputed. Prosthesist usually measured the side that had not amputed and design the socket accordingly without considering the desired length and weight [16].

The myoelectric prosthetic is the most functional devices for an amputee. The prosthetic systems require a combination of electronic and mechanical engineering depending on the extent of functionality required for the device [13]. Instead of using body power and a lot of force to generate motion, myoelectric prostheses make the patient feel like he or she is generating the same nerves to move [13].

The air splint socket system basically uses a FSR pressure sensor [30], which is placed on the surface of the air splint socket, to transfer any pressure detection data to the microprocessor and microcontroller-based system as the input data. The FSR pressure sensor is one of the most accurate and reliable measurement tools available to determine any contact pressure between the residual limb and the socket surface [30]. With the air splint system, the patient does not need to worry about changing the socket size and fitting, since the socket will change the size and fit accordingly within the desired contact of the residual limb.

Although a few studies have been discussed about the biomechanics for elbow, but no researchers has previously examined a biomechanics of elbow for prosthetics user. This paper presents the pressure analysis applied using three different type of prostheses, the kinematics of elbow motion using three different type of prostheses, and the force required to make the prosthetics socket attached and well fitted to the residual limb. The paper will discuss the influences of muscle to the elbow and also the criteria of different type of prostheses that lead to determine the force applied at both upper and lower part of elbow, which taken the elbow as the origin of axis.

## Methods

### Kinematic data

A total of seven transhumeral amputees (7 males) participated in this study. All the subjects were selected from the University Malaya Medical Centre (UMMC), Kuala Lumpur. The inclusion criteria consist of a minimum 12 cm residual limb length (from the shoulder-transhumeral bone to the end of residual limb), no wound and ulcers in the residual limb, and the ability to flexion/extension of shoulder without the use of assistive devices. The subjects were also considered for participation if they had used prosthesis in the last two years. All human test protocols were approved by the University of Malaya Medical Centre Ethics committee, and each subject’s written, informed consent was obtained before data collection.

### Experimental setting and procedures

Two F-Socket sensors arrays 9811E were attached to the residual limb. The sensor arrays were positioned on the anterior, posterior, medial and lateral aspects of the residual limb. The posterior sensor was positioned approximately 1 cm above the posterior trim line of the socket. Each sensor was trimmed to fit to the residual limb shapes. This sensor arrangement provided a pressure map that covered 90% of the residual limb. Tekscan software version 6.51 was used to record the interface pressure.

The process of equilibrating the sensor is where the whole sensor point shares the equal amount of pressure to ensure that all 96 senses have a common output. The F-Socket was put into a pressure bladder in order to ensure that each area on the F-Socket had the similar criteria. The sensor was placed in the middle of the bladder and then was subjected to a pressure of 100 kPa by taking the specifications from the manufacturer.

After the process was completed, the sensor was then attached to the amputee residual limb so that the position of the sensor was stable. Silicone liners were used for all sockets, which require no reattachment when changing the socket. The sensor was attached by using the spray adhesive, a type of strong glue. As mentioned earlier, only two sensors were required to cover the area of the residual limb. The F-Socket attached only at the part of the humerus bones that were still left. During the installation of the F-socket to the amputee’s upper elbow, the main part was to confirm that the humerus of the upper elbow was well-attached to each sensor. The F-Socket sensor was trimmed horizontally to reduce the length of the sensor. This step was done to accommodate the subjects with shorter limb in order to obtain a tidier sensor placement, as well as to ensure there was no overlapped sensor. After the stockinet was fully fitted into the residual limb, then the socket was fitted into the stockinet. However, the position and the liner of the sensor stability must be validated so that the data collection was not interrupted.

After the amputees were comfortable with the fitting of the socket, the F-socket sensor connects to the portable to collect some data. The value recording has a vulnerable due to the external noise that may occur. This was due to the sensitivity of the sensor and the dimensions that were physically thin but to be fitted into a small interface space. Some unwanted noises usually occurred because of the bending position for the sensor itself. There were several methods to reduce the noise distraction [30]. The first method is by setting up the noise reduction threshold in the Tekscan’s F-Scan. The value was set up to level 3 so that any values or data below or at this level will be filtered automatically. The second method is by removing any data that were collected without applying the pressure to the sensor. When the F-Scan detected the presence of any data of unmoving pressure, the data may be diminished and the calibration of the sensor was set to zero at that level. The third way to handle this problem is by applying individual measurement to each point of the sensor. Sometimes, one of the sensors gave a high pressure and surrounded by lower pressure points. To make it stable, all of the points can detect using the F-Scan and assigned to be in a level position to each other. Therefore, the data of pressure on the interface socket can be collected precisely and correctly.

### Anthropometry

The uses of anthropometry are to study the physical measurement of the human body by classifying them into few classifications such as sex, weight, height and age. Most of these needs are satisfied by basic linear, area and volume measures [30, 31]. However, human body motion usually requires more specific data such as the torque, force, angular velocity and man power.

The mass for the transradial segment which is the amputed side of this research can be calculated by multiplying the total mass of the human body with 0.00160 according to the anthropometry theorem [30, 31]. The participants’ demographic information, weight, required mass (for the transradial part) and theoretical force applied are shown in Table 1.

**Table 1:** **Age, weight, transradial mass of each subjects and theoretical force calculated**

Subjects	Age	Weight, W = mg, (g = 9.81)	Transradial mass, m (W × 0.0160)	Force (N) F = mg (Theoretical)
1	42	62	0.992	9.73152
2	45	73	1.168	11.45808
3	30	59	0.944	9.26064
4	33	77	1.232	12.08592
5	30	80	1.28	12.5568
6	40	72	1.152	11.30112
7	37	62	0.992	9.73152

### Force and torque acting about the elbow joint

The elbow joint remained the origin of axis in this study. The applied force and load to the below elbow part are the same force applied to the upper part of elbow for the normal human hand joint [1–4]. The loads were calculated, and their effects on the small bones and joint surface areas were determined. It is found that the tissues are stressed (which relates to force per unit area) to equivalent levels as those of the lower limb. From this, it follows that the load bearing tissues, such as ligaments and tendons, have similar material properties in the upper and lower limbs [21, 25, 26].

**Figure 2:**
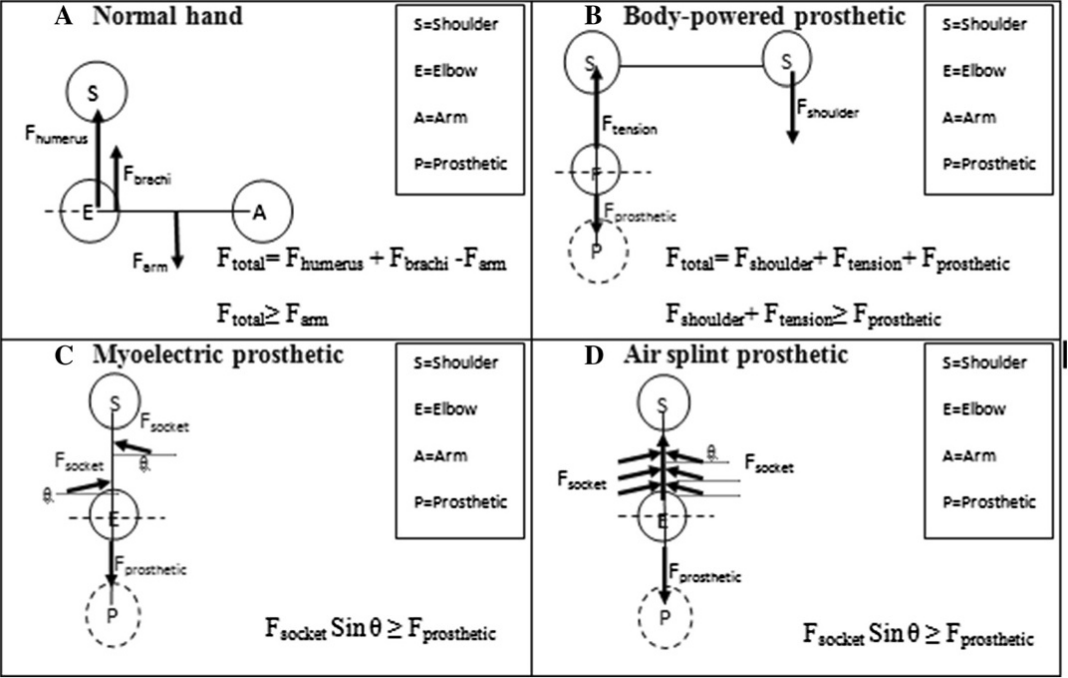
**Comparison of free body diagram from three different types of prostheses; B. Body-powered prosthetic, C. Myoelectric prosthetic, D. Air splint prosthetic and A. normal human hand.** (S= shoulder, E= elbow, A= arm and P= prosthetic). The forces direction react referring to the x-axis and y-axis.

The annual daily life activities (ADL) involve the length and slenderness of the upper limb mean that act at a large distance from the axes of rotation of the joints, and so have a sizeable lever arm [12–14]. Against this are set the upper limb muscles, which must hold a posture or move the hand against an external load known as F_biceps_ in this study. The muscles themselves were all acting at very small movement arms about the joint axes’ origin (elbow, in this study) and thus act at a great mechanical disadvantage [12–14]. Their tensions must be scaled up greatly, in order to attain equilibrium across the joint, known as F_humerus_. As a result of this the joint forces (F_total_) will be much larger than the external loads, F_arm_ and the majority of the forces will be caused by the internal muscle tensions, and not by the relatively small external load [21, 25, 26]. Equation below determines how the total force occurs at the elbow joint for normal human hand (Figure 2A).
12

The torque applied at the elbow joint for normal hand determine by,
3

The summation of torque at the elbow for normal hand equals to zero by taking the elbow joint as the origin. The force and torque applied for body-powered prosthesis are differed according to the mechanism of the prosthetic that using tension cable and shoulder power of a human body. The force applied by the shoulder is to maintain and counter the force required to generate the body–powered prosthetic known as F_shoulder_. The force of the prosthetics itself known as F_prosthetic_ consider as the force for arm (F_arm_) if compared to the normal hand that previously mentioned. F_tension_ is the force applied by the tension cable of a body-powered prosthesis. Equation below determines how the total force occurs at the elbow joint for body-powered prosthesis (Figure 2B).

45

The torque applied at the elbow joint for body-powered prosthetic determine by,
6

The force and torque applied for both myoelectric prosthesis and air splint prosthesis, however, different according to the mechanism of the prosthetics. F_prosthetic_ considers as F_arm,_ occur at the elbow joint balanced by the force applied by F_socket_. F_socket_ gives the resultant force that applied to make sure that the socket of the prosthetic is fully fit and stable to be used by the amputee. It noted that the F_socket_ for both myoelectric and air splint prostheses need to be greater or equal to the weight of the prosthetic itself, otherwise the prosthetic will be loose accordingly. Equation below determines how the total force occurs at the elbow joint for both myoelectric and air splint prostheses (Figure 2C and D).
78

The torque applied at the elbow joint for myoelectic and air splint prostheses determine by,
9

### Equation of force

The F-Scan sensor for this experiment provides the resultant of pressure applied to the socket of the prosthetics. The force can be determined from the resultant of pressure for each three type of prosthetics by dividing the pressure contact with the area of the socket that attached to the residual limb. Equation below identifies how the resultant of pressure from the experiment can determine the force applied in order to hold the prosthetics to the residual limb.
1011

For the body-powered and myoelectric prostheses, the area of pressure involves is fixed since the pressure applied is at a constant place, but the value may change accordingly when motion or load is applied [16, 30]. For air splint prosthesis the area of pressure involved may be changed accordingly to the required size and fit the socket since the pressure was compressed and released automatically by the socket systems. This was explained by the authors in previous studies [16, 30]. Equation below determines how the resultant of pressure from the experiment can determine the force applied using the air splint prosthesis in order to hold the prosthetics to the residual limb.
12

The resultant force determines from the experiment react as the F_shoulder_ + F_tension_ for body-powered prosthesis and F_socket,_ for both myoelectric and air splint prostheses (Refer Figure 2).

## Results

The results of pressure applied using three types of the prosthetics system shown in Table 2. Subject 3 has the minimal criteria of age, and body weight compare to other subjects and turnout given the least pressure applied to the stump socket with a body-powered prosthesis = 4.32 kPa, myoelectric prosthesis = 5.72 kPa, and air splint prosthesis = 2.91 kPa. The maximum result of the pressure applied to the stump using all three types of prosthetics came from subject 5 which sharing the same age as subject 3 but have the maximum body weight. The results for this subject are; body-powered prosthesis = 8.21 kPa, myoelectric prosthesis = 8.01 kPa, and air splint prosthesis = 5.97 kPa. Table 2 also includes the area of pressure applied. The area for body-powered prosthetic is about 0.03 ± 0.005 m while 0.045 ± 0.005 m for both myoelectric and air splint socket systems. Note that the area for body-powered cover static area and at a different place which came from the socket and insole design [16, 30, 32].

**Table 2:** **Result pressure profile applied for each type of prostheses**

Subjects	Body-powered socket pressure, kPa	Myoelectric socket pressure, kPa	Air splint socket pressure, kPa
	Area (0.03±0.005 m)	Area (0.045±0.005 m)	Area (0.045±0.005 m)
1	5.78	6.02	3.22
2	7.47	7.45	5.24
3	4.32	5.72	2.91
4	7.35	7.62	5.61
5	8.21	8.01	5.97
6	6.58	6.48	4.93
7	6.21	6.09	4.23

## Discussion

Different prosthetics applied different kinematics mechanism [28]. As for body-powered prosthetics the kinematics involved both part of shoulder. Any motion to be generated is dependable to the shoulder power, including holding the prosthetics to the residual limb [19]. The socket design and fitting for this type of prosthetics also provide the force to make sure the socket is well attached to the residual limb. The resultant force will be balance if the shoulder force and socket force applied is equivalence or greater than the force given by the body-powered prosthetics (Figure 2B) [19, 28]. The statically design of a body-powered socket, give the statically area for pressure applied to the residual limb. The pressure occurs to be very high and become higher by the time the subject provides any motion. The weight of the prosthetics itself is also too heavy and need a lot of force in order to hold it to the residual limb [31, 33]. According to anthropometry [16, 30, 32], the force applied for normal hand is about 9 N.m-12 N.m but the result for body-powered prosthesis is about 12 N.m-22 N.m. (Refer Table 3).

**Table 3:** **Comparison of force applied by each type of prostheses with the normal hand force**

Subjects	Theoretical force F _joint_ (N)	Body-powered F _tension_ +F _shoulder_ (N)	Myoelectric F _socket_ (N)	Myoelectric F _socket_ (N)
			(static area)	(dynamic area)
1	9.73152	17.34	27.09	14.49
2	11.45808	22.41	33.525	23.58
3	9.26064	12.96	25.74	13.095
4	12.08592	22.05	34.29	25.245
5	12.5568	24.63	36.045	26.865
6	11.30112	19.74	29.16	22.185
7	9.73152	18.63	27.405	19.035

For the myoelectric prosthesis, the resultant force applied is around 25 N.m -36 N.m. The joint force may be even higher than shown in Table 3, because there is also electromyography evidence of antagonistic triceps activity during elbow motion, acting to stabilize the joint [16–19]. The area that applied pressure is dynamically changed in bigger shape compared to the body-powered prosthetics [19, 30]. The pressure applied to the socket must be greater and dynamically change dependable to the weight of the prosthetics device [27–29]. Generally known, the myoelectric prosthesis consists of motors, microcontroller, cable and power supply, which gives heavy load to the design of the myoelectric prosthesis. The socket design and fitting for the myoelectric prosthesis must also be equivalence or greater than the weight of the prosthetics itself (Figure 2C). Abd Razak et al. [30] described on how the pressure applied to the prosthetics socket play a major part to provide the force to hold and fit the socket to the residual limb. However, the socket size and fitting for this type of prosthesis remain unchanged which can cause discomfort among the users. The interface of a socket to the residual limb brings discomfort to the user. The load applied wearing the myolectric together while generating the motion also brings discomfort to the user [19, 27–29]. Some user refuse to be worn it for more than 2 hours to doing daily life activities (ADL) [16, 30]. Previous studies have shown that load compliance can influence the time failure during sustained contractions at low target forces, but not when the target force was similar to the upper limit of motor unit recruitment [20–25].

For the air splint prosthesis, the resultant force applied is around 14 N.m-26 N.m. Even though the systems using the almost the similar part as the myoelectric prosthesis, but the dynamically change of the socket make the pressure applied more reliable to use it (Refer Figure 2D). The socket for air splint prostheses is changeable to the load applied [16, 30]. The pressure applied by the air splint socket will change accordingly to the need in order to hold it to the residual limb. By using this mechanism, the pressure will increase if a greater load applied by the prosthetics. Even the force applied may be greater than the desire force for normal human hand, but the applied value is still relevance and change accordingly. The clinical importance of these biomechanical aspects relates not only to the size of the loads on the elbow but also the directions and points of application of the loads [1, 2, 5]. This principle reacts as the same principle for our upper limb muscle, which reacts if a greater load applied to generate any motion [6–11]. While load sharing clearly suggests the mechanical logic for this approach, there is no prosthesis currently available that appears to achieve reliable long-term outcomes.

Muscle activities always changeable whenever a load is applied [11]. For this study, elbow is the origin of the axis. Meaning that whenever load applied to the below elbow part, the muscle tension formed all muscle of upper elbow (brachiallis, biceps brachii and brachioradialis) will react [1–4]. This is a difficult situation to analyze mechanically because of the large number of muscles acting simultaneously, and so a scheme for apportioning the muscle actions is needed [6–11]. For prosthetics user, the transhumeral amputee loses their elbow and replaced by prosthetics elbow as the axis in this study. The muscle may be active, and some may be inactive due to the amputed [27–29]. For the body-powered prosthesis, both conditions can be considered as there will be no requirement to generate a motion from active muscle [29]. The case is different for both myoelectric prosthesis and air splint prosthesis where the active muscle is needed to generate the motion. That is why by using a different type of prostheses, the rehabilitation is still needed from time to time in order to train and straighten the muscle [28].

The condition of the prosthetics itself also contributed to a major part of generating the biomechanics movement for elbow. Considering the different material of socket manufacturing, the socket may be loose easily from time to time according to the load applied [16, 30]. The material used may easily make the user sweat and cause pain, which may loosen the prosthetics that attaching to the residual limb. The loosen prosthetics socket may interrupt the motion, and force applied at the elbow. The conditions of both body-powered and myoelectric are same, where the socket is static and provide a different pressure considering the body weight is changing daily [28]. For the air splint prosthesis, the socket size is changeable with the required size for the residual limb. With this system, the pressure and force applied is changing accordingly to the need and would not interrupt the motion of elbow [16, 30].

Table 3 shows the comparison of force applied from all seven subjects using three different type prostheses. The table shows that the higher force applied when the subjects using the myoelectric prosthesis. The force applied to both body-powered and air splint prostheses are quite similar, but the force and pressure come at different aspect and criteria. The air split prosthesis design considering the biomechanics principle that applied when involving any motion at the elbow. Besides arm, flexion and extension of elbow play a major contribution to load a thing [12–14]. Different prosthetics applied different biomechanics in order to maintain the socket to be attached to the residual limb [19]. The air splint counters the static socket problem by providing the auto sizing socket that leads to the required pressure applied to the socket, to make sure the socket is well fitted, suitable and comfortable to the user. For the statically socket, the principle is using the socket to give pressure to the residual limb. The result shows how both applied too much pressure that needs to strengthen the socket holding to the residual limb. Besides, this brings the reshaping of the residual limb based on statistic pressure position of the prosthetics socket. The weight of prosthetics, daily change of body weight and generating a motion contributed to the rejection of using those types of prosthetics [27–29]. For the air splint prosthesis, the socket sizes give the pressure accordingly to the need of holding it to the residual limb. The constant pressure provided constantly surrounding the socket make the user feel more comfortable.

## Conclusion

The elbow is a complex and interesting structural mechanism. The comparison of biomechanics of an elbow joint for three different type of prosthetics with the normal hand bring the new information for the amputees and prosthetist to choose the most suitable device to be worn daily. The force and torque applied at the elbow joint by wearing the prosthetics can help improve the design and rehabilitation procedure. The pressure applied to the socket can determine the future shape and figure of the residual limb. The socket may need to be changed from time to time because of the imbalance of force and pressure that applied to the prosthetics at the elbow and socket systems.
